# The UK Myotonic Dystrophy Patient Registry: facilitating and accelerating clinical research

**DOI:** 10.1007/s00415-017-8483-2

**Published:** 2017-04-10

**Authors:** Libby Wood, Isabell Cordts, Antonio Atalaia, Chiara Marini-Bettolo, Paul Maddison, Margaret Phillips, Mark Roberts, Mark Rogers, Simon Hammans, Volker Straub, Richard Petty, Richard Orrell, Darren G. Monckton, Nikoletta Nikolenko, Aura Cecilia Jimenez-Moreno, Rachel Thompson, David Hilton-Jones, Chris Turner, Hanns Lochmüller

**Affiliations:** 1 John Walton Muscular Dystrophy Research Centre, Institute of Genetic Medicine, Newcastle University, Newcastle upon Tyne, UK; 2 Department of Neurology, RWTH Aachen University, Aachen, Germany; 3 Department of Neurology, Queen’s Medical Centre, Nottingham, UK; 4 Department of Rehabilitation Medicine, Derby Teaching Hospitals NHS Foundation Trust, Derby, UK; 5 Department of Neurology, Salford Royal NHS Foundation Trust, Salford, UK; 6 Institute of Medical Genetics, University Hospital of Wales, Cardiff, UK; 7 Wessex Neurological Centre, University Hospital of Southampton, Southampton, UK; 8 Department of Neurology, NHS Greater Glasgow and Clyde, Southern General Hospital, Glasgow, UK; 9 Department of Neurology, Royal Free Hospital, London, UK; 10 Institute of Molecular, Cell and Systems Biology, College of Medical, Veterinary and Life Sciences, University of Glasgow, Glasgow, Scotland, UK; 11 Department of Clinical Neurology, John Radcliffe Hospital, Oxford, UK; 12 UCL MRC Centre for Neuromuscular Diseases, Institute of Neurology, London, UK

**Keywords:** Myotonic dystrophy, Patient Registries, Clinical trials, Trial readiness

## Abstract

Myotonic dystrophy type 1 (DM1) is the most frequent muscular dystrophy worldwide with complex, multi-systemic, and progressively worsening symptoms. There is currently no treatment for this inherited disorder and research can be challenging due to the rarity and variability of the disease. The UK Myotonic Dystrophy Patient Registry is a patient self-enrolling online database collecting clinical and genetic information. For this cross-sectional “snapshot” analysis, 556 patients with a confirmed diagnosis of DM1 registered between May 2012 and July 2016 were included. An almost even distribution was seen between genders and a broad range of ages was present from 8 months to 78 years, with the largest proportion between 30 and 59 years. The two most frequent symptoms were fatigue and myotonia, reported by 79 and 78% of patients, respectively. The severity of myotonia correlated with the severity of fatigue as well as mobility impairment, and dysphagia occurred mostly in patients also reporting myotonia. Men reported significantly more frequent severe myotonia, whereas severe fatigue was more frequently reported by women. Cardiac abnormalities were diagnosed in 48% of patients and more than one-third of them needed a cardiac implant. Fifteen percent of patients used a non-invasive ventilation and cataracts were removed in 26% of patients, 65% of which before the age of 50 years. The registry’s primary aim was to facilitate and accelerate clinical research. However, these data also allow us to formulate questions for hypothesis-driven research that may lead to improvements in care and treatment.

**Electronic supplementary material:**

The online version of this article (doi:10.1007/s00415-017-8483-2) contains supplementary material, which is available to authorized users.

## Introduction

In most populations myotonic dystrophy is the most common muscular dystrophy. With an estimated prevalence of 10 per 100,000 people affected in European populations [1, 2], we estimate upwards of 6500 people to be affected in the UK, the majority with myotonic dystrophy type 1 (DM1). DM1 is one of the most variable human diseases with complex, multi-systemic, and progressively worsening symptoms. The main muscle symptoms are distal to proximal muscle weakness and myotonia. Pulmonary and cardiac functions are also impaired with sudden death from cardiac complications being a significant cause of fatality [3]. Other prominent clinical features are cataracts, cognitive and intellectual deficits, endocrine abnormalities, and gastrointestinal related symptoms [4]. Excessive daytime sleepiness and psychiatric symptoms might lead to restricted social participation and quality of life can be seriously impaired [5–7]. Some gender differences for phenotype severity in DM1 have been suggested recently in large French cohort study [8]. There is currently no curative treatment for this complex condition.

DM1 is the result of a triplet CTG repeat expansion in the 3′-untranslated region of the *dystrophia myotonica*-protein kinase (*DMPK*) gene on chromosome 19 [9–11]. The diverse range of symptoms is thought to occur due to the ongoing expansion of the repeat throughout life in different organs, which results in aberrant splicing in a large number of transcripts [12, 13]. The better understanding of the underlying molecular pathology has led to the design of new targeted treatment approaches such as antisense oligonucleotide therapies [14–16] and has increased commercial and academic interest in DM1 over recent years. As scientific and clinical progress is made, it is paramount to prepare this diverse population for clinical trials and patient registries can be a useful and important tool in helping to overcome many of the hurdles faced by rare diseases. Notably they can be utilised for planning and recruitment of clinical trials [17–21].

The UK Myotonic Dystrophy Patient Registry was established in May 2012 with support from the Muscular Dystrophy UK (MDUK) and the Myotonic Dystrophy Support Group (MDSG), assisted by the TREAT-NMD Alliance (www.treat-nmd.eu). The registry is coordinated from the John Walton Muscular Dystrophy Research Centre at Newcastle University and collects clinical and genetic information about both DM1 and myotonic dystrophy type 2 (DM2). A total of 610 patients enrolled by July 2016, here we report the demographic and clinical findings for the 556 symptomatic DM1 patients registered.

## Methods

### Design and setup

The TREAT-NMD Alliance is the result of a EU-funded network of excellence with the remit of “reshaping the research environment” in the neuromuscular field [17]. The dataset collected within the registry includes all mandatory and highly encouraged items agreed at the 2009 TREAT-NMD and Marigold Foundation workshop held in Naarden [18] (Table 1, full questionnaire available in supplementary material).

**Table 1:** Data items collected in the UK Muscular Dystrophy Patient Registry, defined as patient or professional reported

Patient-reported data items	Professional-reported data items
Demographics	Age of onset
Family history	Genetic confirmation (date, laboratory, method, repeat number)
Ethnic origin	Medication
Ambulatory status	Heart condition (including cardiac implant)
Wheelchair use	Electrocardiogram
Myotonia (including medication)	Ventilation
Fatigue/daytime sleepiness (including medication)	Forced vital capacity (%)
Dysphagia	Gastric tube
Pregnancy	Cataract

The UK Myotonic Dystrophy Patient Registry allows the patient to initiate registration and provide the majority of the information themselves online; while enabling them to nominate a healthcare specialist (clinicians, nurse specialists and physiotherapists) who, using a separate online account, enters clinical and genetic details. This includes professionals from secondary healthcare only and does not include general practitioners (GPs). This bespoke system allows the healthcare professional to amend any inconsistencies in the patient data, while providing the necessary clinical and genetic details. This has been tested through a compliance check performed on a random sample of 26 patient records (Newcastle 8, Oxford 10, London 8) which showed a high level of consistency between patient and clinician data across three of the patient-reported symptoms (ambulatory status 100%, dysphagia 100% and myotonia 84%).

Participants are encouraged to update details annually allowing for the collection of longitudinal data. Informed consent is provided online and allows for future contact to be made, data provided to be used in research, and for the additional data to be entered by a nominated professional. The registry has received full ethical (Newcastle and North Tyneside 1 11/NE/0179) approvals for conducting these activities in the UK. The operation and maintenance of the registry is carried out by a part-time curator at an annual budget of approximately £15,000. It is governed by a steering committee comprising multiple stakeholder groups who adhere to Terms of Reference and Standard Operating Procedures (available in supplementary material, details of the committee are available on the registry website http://www.dm-registry.org/uk).

Engagement and participation are known challenges in this population [6, 22]. Therefore, recruitment has involved a number of channels including working closely with patient support and advocacy groups MDSG and MDUK. In addition to distributing leaflets and newsletters, the regsitry engaged with molecular genetic diagnostic laboratories diagnosing the condition and encoraged them to include a statement about the registry on all positive diagnostic reports.

### Data cleaning and analysis

The completeness of both patient reported and professional-reported data varies, leading to different denominators in the calculations of percentages, which is clearly stated in the results. Where more than one-time point is available for any data item the most recent complete entry has been used. A positive genetic diagnosis was considered when the genetic report states that there is a CTG triplet repeat expansion in the affected range at the DM1 locus. Where a genetic diagnosis was not available the clinical or patient self-report diagnosis has been taken into account. Age of onset has been determined based upon the age provided by the treating clinician and was classified into five clinical forms, as recently proposed by Dogan et al. [8]: (1) congenital form, onset from birth to 1 month; (2) infantile form, onset from 1 month to 10 years; (3) juvenile form, onset at 11–20 years; (4) adult form, onset at 21–40 years; and (5) late adult form, onset after the age of 40 years.

### Statistical methods

Statistics have been carried out using IBM SPSS statistics 22. Correlation between two ordinal variables (e.g. severity of symptoms) has been calculated using Spearman’s *ρ*. Chi-square test and if appropriate Fisher’s exact test were applied in categorical variables to determine statistical significance. *p* values less than 0.05 were considered to be statistically significant.

## Results

**Fig. 1:**
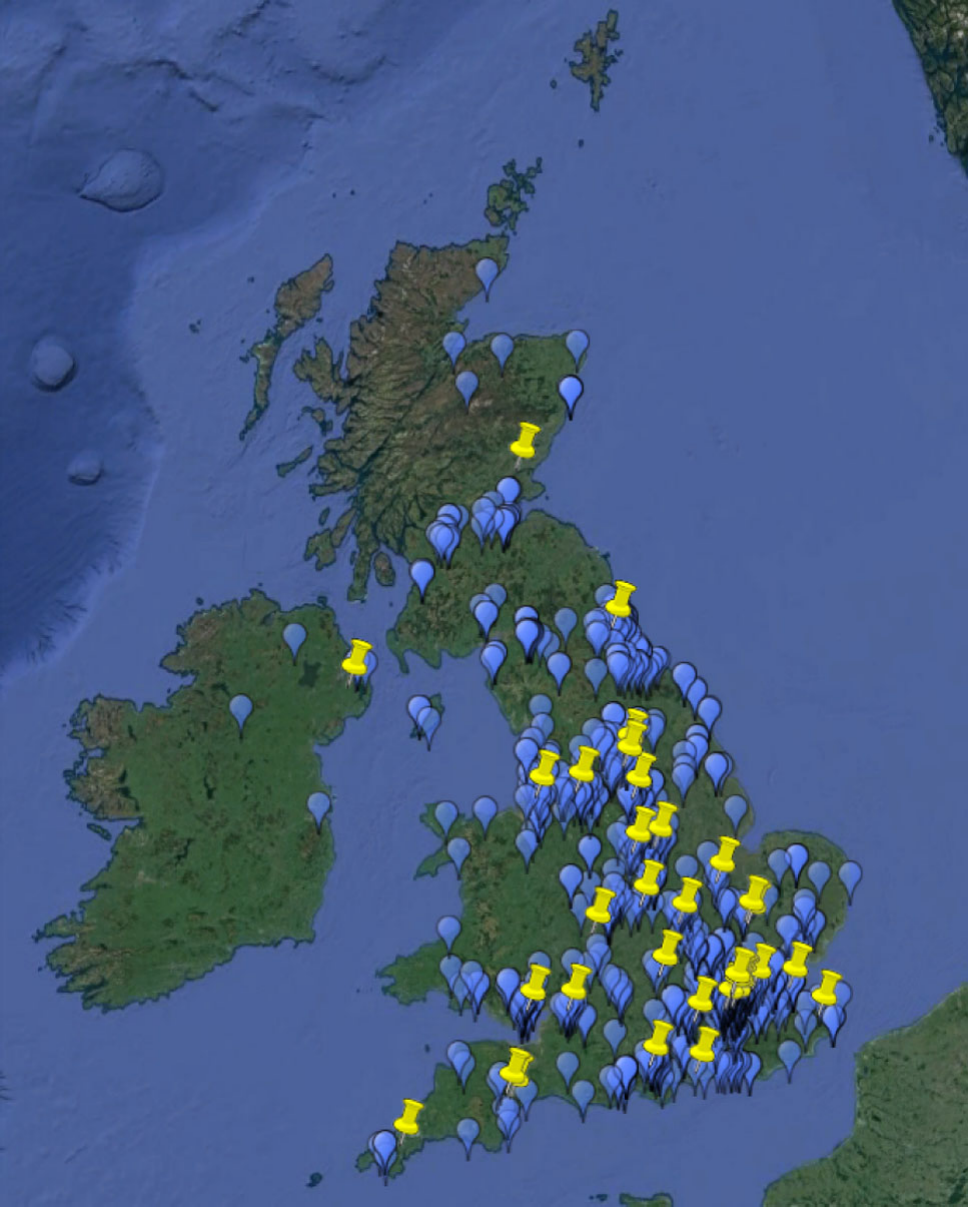
Map of patients and doctors of the UK Muscular Dystrophy Patient Registry. *Blue pins* represent an individual patient and *yellow pins* a doctor providing data

**Fig. 2:**
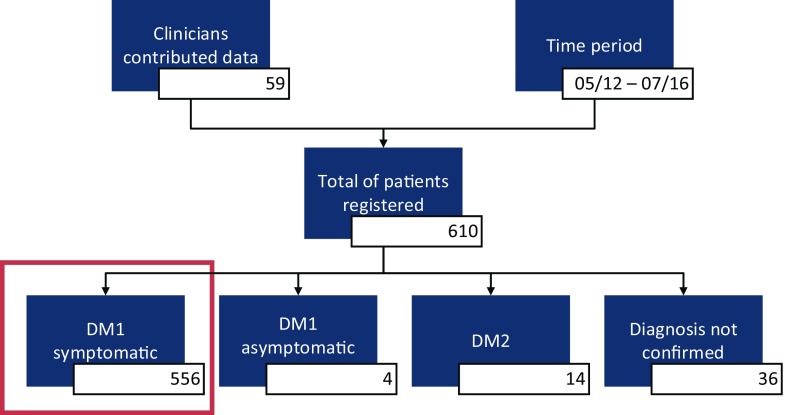
Selection of patients for the UK Muscular Dystrophy Registry Study. *Red rectangle* highlights the patients selected for the study

A total of 610 patients registered in the UK Myotonic Dystrophy Patient Registry between May 2012 and July 2016. A steady increase in registrants has been observed with the largest growth in numbers seen when the registry launched. On average 13 patients registered each month. Sixty-seven healthcare professionals (clinicians, specialist nurses and physiotherapists) have engaged and agreed to contribute data to the registry (Fig. 1). Of all 610 patients, 556 reported DM1 with symptoms, 4 were asymptomatic, 14 reported DM2, and 36 had yet to receive a confirmed diagnosis (Fig. 2). The following analysis included only the symptomatic DM1 patients.

### Patient demographics

**Fig. 3:**
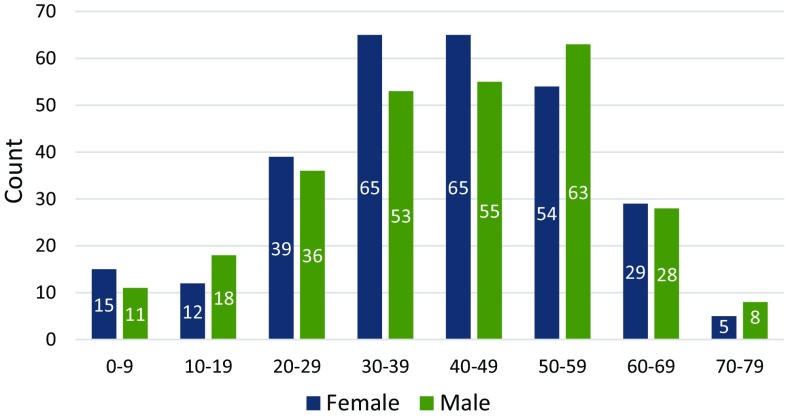
Age range of patients at the time of registration stratified by gender. A broad range of ages was present from 8 months to 78 years with the largest proportion between 30 and 59 years

A positive family history of the condition was reported by 89.1% (475/533) of patients although the detail of the family relationship between affected persons was not collected. Caucasian ethnic origin was reported by 97.3% (532/547) of registrants, Asian by 0.009% (5/547), Black African by 0.002% (1/547) and mixed by 0.02% (9/547). A wide geographical distribution was seen with some clustering around the large neuromuscular centres in Newcastle, Oxford, and London (Fig. 1). An even distribution was seen between genders (women 51.1% (284/556), men 48.9% (272/556)) and a broad range of ages was present from 8 months to 78 years (mean 41.1, standard deviation (SD) ± 16.5) with the largest proportion, 63.8% (355/556) between 30 and 59 years (Fig. 3). The age of onset was available for 32.7% (182/556) of all patients and ranged from birth to 68 years (mean 25.6, SD ±15.9), with the largest proportion having adult onset (Table 2).

**Table 2:** Frequency of symptoms and characteristics stratified by gender and age of onset

	Male 48.9%(272/556)	Female 51.1% (284/556)	Congenital 8.8% (16/182)	Infantile 5.5% (10/182)	Juvenile 27.5% (50/182))	Adult 39% (71/182)	Late adult 19.2% (35/182)	Total *n* = 556
Myotonia, % (No.)	82.9 (218/263)	73.1 (204/279)	68.8 (11/16)	80 (8/10)	89.4 (42/47)	78.3 (54/69)	54.3 (19/35)	77.9 (422/542)
Severe	26.1 (57/218)	17.6 (49/204)	9.1 (1/11)	12.5 (1/8)	23.8 (10/42)	25.9 (14/54)	36.8 (7/19)	25.1 (106/422)
Fatigue, % (No.)	79.6 (215/270)	78.6 (217/276)	75 (12/16)	90 (9/10)	83.7 (41/49)	72.1 (49/68)	74.3 (26/35)	79.1 (432/546)
Severe	23 (62/270)	32.2 (89/276)	8.3 (1/12)	11.1 (1/9)	39 (16/41)	46.9 (23/49)	23.1 (6/26)	35 (151/432)
Dysphagia, % (No.)	51 (132/259)	45.5 (122/268)	33.3 (5/15)	33.3 (3/9)	44.9 (22/49)	34.4 (22/64)	62.5 (20/32)	48.2 (254/527)
Ambulatory assisted or non-ambulant, % (No.)	35.2 (94/267)	34.9 (98/281)	31.3 (5/16)	30 (3/10)	26.5 (13/49)	37.1 (26/70)	31.4 (11/35)	35 (192/548)
Wheelchair use, % (No.)	26.8 (72/269)	25.5 (71/278)	43.8 (7/16)	20 (2/10)	20 (10/50)	26.1 (18/69)	22.9 (8/35)	26.1 (143/547)
Heart condition, % (No.)	50.9 (58/114)	45.5 (50/110)	30.8 (4/13)	50 (5/10)	46.9 (23/49)	51.4 (36/70)	53.1 (17/32)	48.2 (108/224)
Non-invasive ventilation, % (No.)	16.2 (19/117)	13.6 (16/118)	12.5 (2/16)	0 (0/10)	10 (5/50)	20 (14/70)	14.7 (5/34)	14.9 (35/235)
Cataract surgery, % (No.)	22.5 (25/111)	29.5 (33/112)	0 (0/16)	0 (0/10)	14.9 (7/49)	36.8 (25/68)	48.6 (17/35)	26 (58/223)

Age of onset has been determined based upon the age provided by the treating clinician and was classified into: (1) congenital form, onset from birth to 1 month old; (2) infantile form, onset from 1 month to 10 years; (3) juvenile form, onset at 11–20 years; (4) adult form, onset at 21–40 years, and (5) late adult form, onset after the age of 40 years

Fractions give the absolute number of patients divided by the number of patients with available clinical information for each item

### Symptoms and treatments

**Fig. 4:**
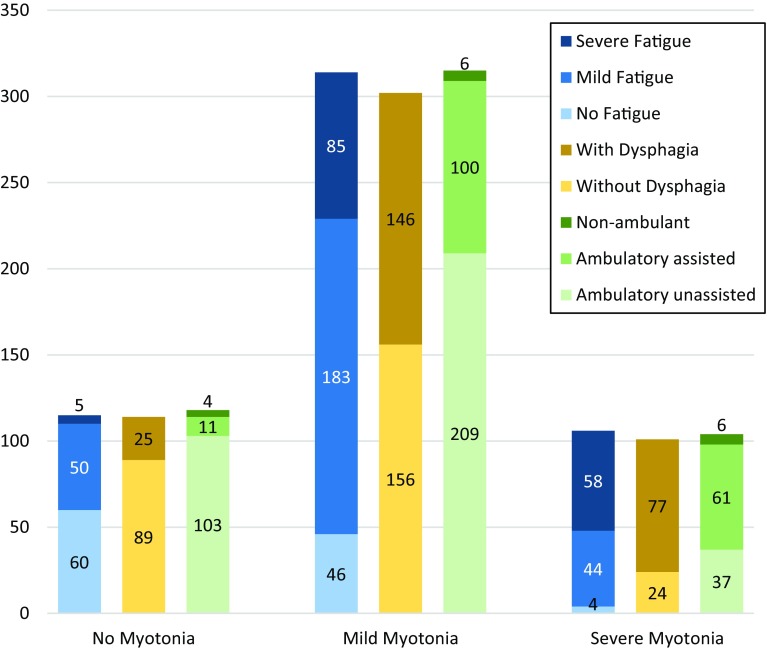
Association between myotonia and the other symptoms fatigue, dysphagia, and ambulatory status The correlation between myotonia and fatigue was statistically significant (*ρ* = 0.461, *p* < 0.001), with relatively more patients having mild fatigue among the patients with mild myotonia, and relatively more patients having severe fatigue among the patients with severe myotonia. Similarly, a correlation between myotonia and ambulatory status existed (*ρ* = 0.337, *p* < 0.001). Dysphagia occurred significantly more frequently in patients with myotonia (*p* < 0.001). *Numbers* refer to patients with available information for the respective symptoms

The most commonly reported symptoms in the registry were fatigue/daytime sleepiness and myotonia, reported by 79.1 and 77.9% of patients, respectively (Table 2). These symptoms were most often described as mild [fatigue 65% (281/432); myotonia 74.9% (316/422)] and occurred across all ages (Table 2). The correlation between myotonia and fatigue was statistically significant (*ρ* = 0.461, *p* < 0.001), with relatively more patients having mild fatigue among the patients with mild myotonia, and relatively more patients having severe fatigue among the patients with severe myotonia (Fig. 4). Similarly, there was a significant correlation between myotonia and ambulatory status (*ρ* = 0.337, *p* < 0.001). Dysphagia occurred significantly more frequently in patients with myotonia (55.3%, 223/403) compared to patients without myotonia (21.9%, 25/114, *p* < 0.001, Fig. 4). No correlation existed between fatigue and ambulatory status (*ρ* = 0.231).

A treatment with modafinil was given in 18.5% (80/432) of patients with fatigue and 7.4% (32/432) used non-invasive ventilation. Medication to help manage myotonia was reported by 6.4% (27/422) of patients with myotonia, with the most common medication mexiletine reported in 40.7% (11/27). Dysphagia was reported in 48.2% of patients (Table 2), but only 2 patients with dysphagia used a gastric tube for feeding.

The majority of patients, 65% (356/548), were ambulant not using any assistive device to walk, 31.8% (174/548) required some assistance, and 3.3% (18/548) were non-ambulant. Of the patients needing some assistance, wheelchair use was reported by 56% (94/168) of patients, including 95.7% (90/94) of them using a wheelchair part-time and 4.3% (4/94) using a wheelchair most of the time. The age at which wheelchair use began ranged from less than 1 to 72.4 years (mean 34.6, SD ±21.6). Among the wheelchair users 22.8% (32/129) started before the age of 10 years and 56.6% (73/129) started to use a wheelchair between 30 and 59 years.

### Cardiac and pulmonary function

A routine electrocardiogram was reported for 33.8% (188/556) and a heart condition (arrhythmia or conduction block) was reported in 48.2% of patients (Table 2). The age at diagnosis of heart condition ranged from less than 1 to 76.4 years (mean 36.5 years, SD ±19.1), with 73.6% (78/106) of patients showing an onset before the age of 50 years and 15.1% (16/106) having a heart condition before the age of 18 years. A wheelchair was used in 27.4% (29/106) of patients with cardiac abnormalities where information for both items was available. A cardiac implant was carried by 36.1% (39/108) of patients with a heart condition, of which a pacemaker was reported in 87.2% (34/39) and an implantable cardioverter defibrillator (ICD) in 12.8% (5/39) of patients. Information about the age at which cardiac implant was inserted was available for 82.1% (32/39) of patients, 56.3% (18/32) of which required a cardiac implant before the age of 50 years, but no pacemaker was inserted in patients under 18 years of age.

Regular and ongoing non-invasive ventilation was needed in 14.9% of patients (Table 2), 52.9% (18/34) of which reported mild and 41.2% (14/34) severe fatigue. Of those using non-invasive ventilation, 64.7% (22/34) required some assistance when walking and 50% (17/34) used a wheelchair at least part time. Patients using non-invasive ventilation reported a heart condition significantly more frequently (76.7%, 23/30) compared to patients without non-invasive ventilation (43.2%, 83/192, *p* = 0.001). Seventeen percent (6/35) of patients with ventilation were under the age of 30 years and there was no one registered with invasive ventilation. The age at which ventilation began was not recorded. The results of pulmonary function testing were reported in 25.9% (144/556) of patients, with 52.8% (76/144) being normal (>70% forced vital capacity (FVC)), 14.6% (21/144) showed moderate (60–69% FVC), 13.9% (20/144) moderately severe (50–59% FVC) and 18.8% (27/144) severe (<50% FVC) restriction. Of those with severe restriction, 22.2% (6/27) of patients reported using non-invasive ventilation, 80% (20/25) reported fatigue and 51.9% (14/27) used a wheelchair at least part time. Of the patients with an FVC of greater than 70%, 5.3% (4/76) were using a wheelchair part time compared to 42.4% (28/66) of those with an FVC below 70%.

### Cataracts

Information about cataract surgery was available for 40.1% (223/556) of patients registered, of which 26% had cataracts removed (Table 2). Age of surgery was available for 84.2% (48/57) of these patients, 31.3% (15/48) of which had surgery before the age of 40, 33.3% (16/48) reported surgery between 40 and 49 years, and the remaining 35.4% (17/48) had surgery after the age of 50 years.

### Genetic diagnosis

The age of genetic diagnosis was available for 33.8% (188/556) of patients and ranged from birth to 72 years (mean 36.4, SD ±15.7). The diagnosis of a large proportion of patients, 65.4% (123/188), took place between 20 and 49 years. There was a notable age gap between the age of symptom onset and the age of genetic diagnosis; 14.9% (20/134) participants received a genetic diagnosis within 12 months of symptoms onset and 13.4% (18/134) had the genetic diagnosis before the age of onset. However, the mean period from onset of symptoms to genetic diagnosis was 9.1 years (SD ±11.1), for those with symptoms before diagnosis the delay was 11.1 (SD ±10.4) years.

The CTG repeat length reported from standard diagnostic testing was only available for 11.3% (63/556) of patients registered and ranged from 21.7 to 867 CTG trinucleotide repeats (mean 364.3, SD ±266.1). Information about age at onset was given for 81% (51/63) of those with a repeat number available, 3.9% (2/51) of which were categorised as congenital onset, 5.9% (3/51) as infantile, 35.3% (18/51) as juvenile, 31.4% (16/51) as adult, and 23.5% (12/51) as late adult onset. The two patients reporting congenital onset had a reported CTG repeat number of 450 and 700, those with infantile onset had repeats between 700 and 833, juvenile onset patients had 129–867 repeats, adult onset 67–700, and repeat numbers of patients with a late adult onset varied from 70 to 700. The mean age at which the genetic testing was performed was 13.5 years for patients with congenital onset, 28.1 years for infantile onset patients, 27.9 years for patients with juvenile onset, 37.3 years for adult onset, and 52.2 years for patients with late adult onset.

The severity of symptoms (myotonia, fatigue, ambulatory status) was not correlated to the repeat size at the time of diagnostic testing.

### Gender differences

Myotonia was reported significantly more frequently by male patients compared to females (*p* = 0.006) and men had significantly higher occurrence of severe myotonia (*p* = 0.021). Fatigue occurred almost as frequently in men as in women (Table 2), but women reported this fatigue as severe significantly more frequently (*p* = 0.028). Although not statistically significant, more male patients reported dysphagia compared to female DM1 patients (*p* = 0.21). There were more male patients with mobility impairments (*p* = 0.84), wheelchair use (*p* = 0.74), cardiac abnormalities (*p* = 0.42), and the need of non-invasive ventilation (*p* = 0.56), whereas more women had cataracts removed (*p* = 0.24), but these differences were not statistically significant.

## Discussion

### Successes and limitations

A diverse group of myotonic dystrophy patients have registered, providing a cross-sectional snapshot of the myotonic dystrophy population in the UK and the contribution from healthcare professionals (clinicians, nurse specialists and physiotherapists) across the country has helped establish a virtual network of medical professionals with an interest in myotonic dystrophy and research into the condition [23]. The registry has successfully supported recruitment into several academic research studies, including the international trial OPTIMISTIC [24] (NCT02118779), the national deep-phenotyping study Pheno-DM1 (NCT02831504), and a pilot longitudinal study at the University of Nottingham. In each case local clinic-based recruitment was supplemented with nationwide registry recruitment, the latter accounting for 30–50% of study participants. In addition, the registry is supporting recruitment and feasibility for the first commercial clinical trial in the UK (NCT02858908). It has also been utilised as a research tool to help the National Institutes of Health (USA) with ongoing research into myotonic dystrophy and cancer, as well being part of an international effort to collect additional information on common adverse events.

There are limitations to a patient initiated registry and the design of this registry does include an element of self-selection, therefore we cannot assume this cohort is fully representative of the entire DM1 population in the UK. The nature of the registry means that inclusion is biased towards those that are able, willing, and interested to participate in clinical research. It may be speculated that this snapshot represents the less severe and more engaged population of the DM1 population in the UK. However, it could be argued that more severely affected patients may engage more with research as there is a greater impact on their daily lives. The reliability of the patient-reported symptoms such as myotonia could also be questioned as these terms may not be accurately understood by patients, this may be a specific concern in DM1 considering the cognitive impairment present in many. However, a limited compliance check across three centres showed the reliability of patient-reported data to be very promising, validating the move towards an increase in self-reported outcomes. Furthermore, evidence suggests that patients gain additional benefit from this level of engagement [25].

Clinical and genetic information is not available for some patients; this is due to some delays in obtaining local R&D approvals and the burden on the professional’s time. Without full integration into the healthcare system or provision of resources at a larger scale it may not be possible to capture complete genetic and clinical information on all patients.

To improve the completeness of the data a larger proportion of the data items could be completed by the patient themselves, particularly relating to the age at onset and questions regarding medications and ventilation. Having more information about how many patients have tried a treatment but failed to tolerate it could help to improve current best practice and warrant future investigation.

### Clinical lessons and hypothesis generation

The registry has already helped to formulate hypothesis for future studies which may lead to better understanding of the condition. For example, the data presented support a recent study indicating gender differences in DM1 [8]. Similar to this study, men had higher frequencies of severe myotonia, mobility impairments, cardiac abnormalities, and non-invasive ventilation, whereas women presented more often with cataracts. Contrasting the results of Dogan et al., the frequency of patients with dysphagia was slightly higher in men than in women. We additionally found significantly more severe fatigue among female DM1 patients, which has not been described before. Although the symptoms in Dogan et al. were professional reported [8], many of the gender differences were confirmed by our patient-reported data in an independent population.

The most prevalent symptom described in the registry is fatigue and excessive daytime sleepiness, known to also be one of the most disabling leading to disability, unemployment, family breakdown, and reduced quality of life [26, 27]. Clinical reports and small studies suggest that stimulating drugs such as modafinil may have a profound, positive effect on excessive daytime sleepiness and quality of life in DM1 patients [6, 22, 26]. However, after review by the European Medicines Agency it was recommended that due to lack of evidence [28] its use in myotonic dystrophy should be ceased. However, the unmet medical need for treatment for fatigue remains strong and potentially new molecules like pitolisant or other stimulant drugs [29], may offer room for future trials seeking innovative solutions for this prevalent symptom.

A correlation between dysphagia and myotonia has been observed in the registry data, with dysphagia occurring more frequently in patients who also report myotonia. This positive correlation is also seen between myotonia and mobility impairment. This is contradictory to the authors’ clinical impression that dysphagia is associated with muscle weakness and is negatively correlated with myotonia. Further clinical investigation may allow us to understand these findings as they could impact care and treatment.

The data presented suggest that there is a significant delay between symptom onset and genetic diagnosis [30]. This has been shown in other cohorts and suggests a need to adopt better guidelines for the identification and diagnosis of DM1. There is rapid progress in the development of new targeted treatments such as antisense oligonucleotide therapies [14–16] and while these studies are treated with appropriate caution, they highlight the need for detailed genetic information in order for this community to be not only “trial-ready” but also “treatment-ready”. Furthermore, delays in diagnosis prevent the best care being provided to patients, such as existing approaches for monitoring cardiac function and symptomatic treatments for fatigue and myotonia. Genetic testing for DM1 by Southern blot analysis of restriction digested genomic was introduced into the UK in the early 1990s shortly after the identification of the gene in 1992 [9–11]. A move was made to triplet-primed PCR after this method was described in 1996 [31]. Our data do not show a reduction of the diagnostic gap in the last 20 years. This suggests that the delay in genetic diagnosis is not due to availability of a genetic test but to other factors. Consideration should be given to assessing the reasons for the delay and the impact this has on patient care and quality of life.

Our data regarding diagnosis could be improved with better characterisation of age of onset and inclusion of presenting symptom. This could help better understand if certain symptoms such as bowel dysfunction (information about which is not currently collected), cataracts, or excessive daytime sleepiness may not be picked up as quickly as muscle weakness or grip myotonia as suggested by previous studies [32, 33]. As most diagnostic laboratories in the UK use the repeat-primed PCR assay, CTG repeat lengths are not part of the diagnostic report, therefore these data are not readily available for most DM1 patients in the UK. This limits interpretation of genotype–phenotype correlations, the value of this should be considered by health care providers considering the potential added value in prognosis and standards of care.

### Future considerations

The registry allows self-reported outcomes to be collected from a large cohort of patients with direct and ongoing access. The results shown here may inform the design of future academic studies into the pathophysiology of the condition and provide relevant information for clinical trials. The collection of longitudinal data over time will provide an additional resource when assessing the progression of the condition.

The common TREAT-NMD dataset is shared by at least 19 registries across 17 different countries (Argentina, Australia, Bulgaria, Canada, China, Czech Republic, Egypt, France, Germany, Italy, Japan, New Zealand, Poland, Serbia, Spain, UK and US). Ideally, all registries would be linked centrally by this common dataset allowing data to be shared across the research community. The TREAT-NMD Alliance has successfully established a global network of national registries for Duchenne muscular dystrophy and spinal muscular atrophy [34, 35] and continues to play an active and important role in coordinating neuromuscular registries globally. The registry is aiming to be part of any future collaborative efforts by TREAT-NMD.

Increased harmonisation and collaboration not only between registries, but also across resources could enable communication between different health care systems in a sensitive and secure manner. This kind of collaboration and data sharing is key to understanding the natural history of these complex and rare diseases and there are number of initiatives in Europe currently looking at ways to achieve this, for example RD-Connect (http://www.rd-connect.eu) [17, 36].

The UK Myotonic Dystrophy Patient Registry is an example of a novel, online-based, cost-effective, and patient-driven registry. Its success can be measured by its continuous growth and utilisation. Although primarily designed to accelerate and facilitate trial recruitment and planning the registry has also provided and interesting and important data characterising the DM1 patient community in the UK.

## Electronic supplementary material

Below is the link to the electronic supplementary material.

Supplementary material 1 (DOC 2832 kb)

Supplementary material 2 (DOC 2848 kb)

Supplementary material 3 (PDF 103 kb)
