# Supplementary material for: The UK Myotonic Dystrophy Patient Registry: facilitating and accelerating clinical research
Source: J Neurol. 2017 Apr 10;264(5):979–88. doi: 10.1007/s00415-017-8483-2 (PMC5413526; doi:10.1007/s00415-017-8483-2)
Supplement: Supplementary file 3 — Supplementary material 3 (PDF 103 kb) [file 415_2017_8483_MOESM3_ESM.pdf]

Core dataset for international DM1 registry – final draft 1 August 2009

	Item	Self-report example
	**Mandatory items**	
**1.**	**Personal data**<br><br>Sex<br>First name<br>Last name<br>Date of birth<br>Address<br>Zip/post code<br>Telephone<br>Email	**Your personal data**:<br><br>Sex:<br>First name:<br>Last name:<br>Date of birth:<br>Address:<br>Zip/post code:<br>Telephone:<br>Email:
**2.**	**Clinical Diagnosis**<br><br>○ Congenital Myotonic Dystrophy<br>○ DM1<br>○ DM1 asymptomatic mutation carrier<br>○ Other<br>○ Unknown	**What is your diagnosis, according to your doctor?**<br><br>○ Congenital Myotonic Dystrophy<br>○ Myotonic Dystrophy Type 1 (DM1)<br>○ Mutation carrier for DM1 without symptoms<br>○ Other<br>○ I don't know
**3.**	**Genetic test result**<br><br>○ DM1 mutation (triplet repeat expansion)<br>○ Other mutation:……………..<br>○ Result pending<br>○ Not tested	**What is your genetic test result?**<br><br>○ DM1 mutation (triplet repeat expansion)<br>○ Other mutation:……………..<br>○ I have been tested but I haven't received the result yet<br>○ I have not been tested
**4.**	**Current best motor function**<br><br>○ Ambulatory (unassisted)<br>○ Ambulatory (assisted)<br>○ Non-ambulatory	**Which of the following options describes the best motor function you are currently able to achieve? (please tick the most appropriate answer)**<br><br>○ I can walk unaided (without an assistive device)<br>○ I can walk with an assistive device (walker, brace, cane, etc)<br>○ I cannot walk
**5.**	**Wheelchair use**<br><br>○ No<br>○ Part-time (age...)<br>○ Full-time (age ...)	**Do you use a wheelchair? (please tick the most appropriate answer)**<br><br>○ No, not at all<br>○ I use a wheelchair part-time (I started at age: ……….)<br>○ I use a wheelchair all the time (I started full-time use at age: ………..)

	Highly encouraged items	
	**MUSCLE**	
**6.**	**Myotonia**<br><br>o Severe<br>o Mild<br>o None	**Does myotonia (cramping, difficulties releasing your grip, etc.) currently have a negative effect on your normal daily activities?**<br><br>o Yes, severely<br>o Yes, but only mildly<br>o Not at all
**7.**	**Myotonia medication use**<br><br>o Yes (specify...)<br>o No<br>o Unknown	**Do you currently take medication to treat or prevent myotonia?**<br><br>o Yes (specify or choose from drop down list)<br>o No<br>o I don't know
	**CARDIAC**	
**8.**	**Heart condition**<br><br>o Yes, not further specified (age...)<br>o Arrhythmia or conduction block (age...)<br>o Cardiomyopathy (age...)<br>o No<br>o Unknown	**Have you been diagnosed with a heart condition?**<br><br>o Yes, not further specified (at age: ….)<br>o Yes, with arrhythmia or conduction block (at age: ….)<br>o Yes, with cardiomyopathy (at age: ….)<br>o No<br>o I don't know
**9.**	**Cardiac implant**<br><br>o Yes, not further specified (age …)<br>o Pacemaker (age…)<br>o ICD (age….)<br>o No<br>o Unknown	**Have you had an operation to implant a device to control/normalize your heart rhythm?**<br><br>o Yes, not further specified (at age: ….)<br>o Yes, a pacemaker (at age: ….)<br>o Yes, a combined cardioverter-defibrillator (ICD) (at age: ….)<br>o No<br>o I don't know
**10.**	**10. ECG**<br><br>ECG done: yes/no/unknown<br><br>Sinus rhythm: yes/no<br>PR interval: ….. ms<br>QRS duration: ….. ms<br><br>Date	**Have you had an electrocardiogram (ECG)?**<br><br>o Yes<br>o No<br>o I don't know<br><br>**If yes, please fill in the ECG results:**<br><br>Sinus rhythm: yes/no<br>PR interval: ….. ms<br>QRS duration: ….. ms<br><br>Date of examination: ………
**11.**	**Echocardiogram**<br><br>Echo done: yes/no/unknown<br><br>LVEF: …%<br>Date	**Have you had an ultrasound of the heart (echocardiography)?**<br><br>o Yes<br>o No<br>o I don't know<br><br>**If yes, please fill in the echocardiography results:**<br><br>LVEF ……………………%<br>Date of examination: ……………

**12.**	**Cardiac medication use**<br><br>o Yes (specify...)<br>o No<br>o Unknown	**Do you currently take any medication to treat or protect your heart (e.g. ACE-inhibitors, beta-blockers, or anti-arrhythmics)?**<br><br>o Yes (specify or choose from drop down list)<br>o No<br>o I don't know
	**PULMONARY**	
**13.**	**Non-invasive ventilation**<br><br>o Full-time<br>o Part-time<br>o None	**Do you regularly use a non-invasive ventilation device?**<br><br>o Yes, all day<br>o Yes, but only part-time (e.g. at night)<br>o No, never
**14.**	**Invasive ventilation**<br><br>o Full-time<br>o Part-time<br>o None	**Do you use invasive ventilation?**<br><br>o Yes, all day<br>o Yes, part-time<br>o No
**15.**	**Pulmonary function testing**<br><br>FVC done: yes/no/unknown<br><br><br>FVC: …%<br>Date	**Have you had pulmonary function testing?**<br><br>o Yes,<br>o No<br>o I don't know<br><br>**If yes, please fill in the results of the test:**<br><br>FVC ....% (predicted value)<br>Date of the test:…………………
	**DIGESTIVE**	
**16.**	**Dysphagia**<br><br>o Yes<br>o No<br>o Unknown	**Do you have difficulty swallowing (food gets stuck in your throat, choking, etc)?**<br><br>o Yes<br>o No<br>o I don't know
**17.**	**Gastric/nasogastric tube**<br><br>o Yes<br>o No<br>o Unknown	**Do you have a tube (gastric/nasal) for feeding?**<br><br>o Yes<br>o No<br>o I don't know
	**OTHER**	
**18.**	**Cataract surgery**<br><br>o Yes (age …)<br>o No<br>o Unknown	**Have you had eye surgery for cataract removal**?<br><br>o Yes (at age: ….)<br>o No<br>o I don't know
**19.**	**Fatigue/sleepiness**<br><br>o Severe<br>o Mild<br>o No	**Does fatigue or daytime sleepiness currently have a negative effect on your normal daily activities?**<br><br>o Yes, severely<br>o Yes, but only mildly<br>o Not at all

**20.**	**Fatigue medication use**<br><br>○ Yes<br>○ No<br>○ Unknown	**Do you currently take any medication to treat or prevent fatigue or daytime sleepiness?**<br><br>○ Yes (specify or choose from drop down list)<br>○ No<br>○ I don't know
**21.**	**Age of onset**<br><br>○ Congenital<br>○ Age …..<br>○ Asymptomatic<br>○ Unknown	**At what age did the first medical problems occur that may be related to your myotonic dystrophy?**<br><br>○ At birth or within the first 4 weeks of life<br>○ At age …..<br>○ I have no symptoms of myotonic dystrophy<br>○ I don't know
**22.**	**Genetic details/repeat size**<br><br>○ date of test<br>○ name of laboratory<br>○ method of testing<br>○ repeat size<br>○ No<br>○ Unknown	**Are details of your genetic test available?**<br><br>○ Yes<br>▪ date of test……<br>▪ name of laboratory<br>▪ method of testing (Southern, PCR, RP-PCR)<br>▪ repeat size: ….. bp<br>○ No<br>○ I don't know
**23.**	**Positive family history**<br><br>○ Yes<br>○ No<br>○ Unknown	**Has anybody else in your family been diagnosed with the same disease?**<br><br>○ Yes<br>○ No<br>○ I don't know
**24.**	**Ethnic origin**<br><br>○ Caucasian<br>○ Black African/African American<br>○ Asian<br>○ Mixed<br>○ Other<br>○ Declined	**How would you describe your ethnic origin?**<br><br>○ White - European origin (Caucasian)<br>○ Black African/African American<br>○ Asian<br>○ Mixed<br>○ Other<br>○ I choose not to answer this question
**25.**	**Other registry**<br><br>○ Yes (specify...)<br>○ No<br>○ Unknown	**Have you signed up for any other myotonic dystrophy registry?**<br><br>○ Yes (if yes, please specify: ……………………..)<br>○ No<br>○ I don't know
